# Hydrothermal Etching Treatment to Rutile TiO_2_ Nanorod Arrays for Improving the Efficiency of CdS-Sensitized TiO_2_ Solar Cells

**DOI:** 10.1186/s11671-016-1236-9

**Published:** 2016-01-12

**Authors:** Jingshu Wan, Rong Liu, Yuzhu Tong, Shuhuang Chen, Yunxia Hu, Baoyuan Wang, Yang Xu, Hao Wang

**Affiliations:** Hubei Collaborative Innovation Center for Advanced Organic Chemical Materials, Faculty of Physics and Electronic Science, Hubei University, Wuhan, 430062 People’s Republic of China

**Keywords:** Quantum dot-sensitized solar cells, Cadmium sulfide, TiO_2_ nanorod arrays, Hydrothermal etching

## Abstract

Highly ordered TiO_2_ nanorod arrays (NRAs) were directly grown on an F:SnO_2_ (FTO) substrate without any seed layer by hydrothermal route. For a larger surface area, the second-step hydrothermal treatment in hydrochloric acid was carried out to the as-prepared TiO_2_ NRAs. The results showed that the center portion of the TiO_2_ nanorods were dissolved in the etching solution to form a nanocave at the initial etching process. As the etching time extended, the tip parts of the nanocave wall split into lots of nanowires with a reduced diameter, giving rise to a remarkable increase of specific surface area for the TiO_2_ NRAs. The TiO_2_ films after etching treatment were sensitized by CdS quantum dots (QDs) to fabricate quantum dot-sensitized solar cells (QDSSCs), which exhibited a significant improvement in the photocurrent density in comparison with that of the un-treated device, this mainly attributed to the enhancement of QD loading and diffused reflectance ability. Through modifying the etching TiO_2_ films with TiCl_4_, a relatively high power conversion efficiency (PCE) of 3.14 % was obtained after optimizing the etching time.

**Electronic supplementary material:**

The online version of this article (doi:10.1186/s11671-016-1236-9) contains supplementary material, which is available to authorized users.

## Background

Recently, quantum dot-sensitized solar cells (QDSSCs) have attracted much interesting research attributed to their unique advantages involving low cost and high theoretical conversion efficiency [1–3]. In typical configuration of QDSSCs, inorganic semiconductor quantum dots (QDs) such as CdS [4–6], CdSe [7, 8], CdTe [9, 10], and PbS [11] were usually used as sensitizer and exhibited huge advantages over organic dyes, such as low cost, high molar extinction coefficient, size-dependent band gap, and multi-exciton generation effect [12–14]. In addition, TiO_2_ semiconductor as the most successful photoanode material was served as a scaffold layer to adsorb QDs and a medium layer to transport a photo-generated electron. Therefore, the specific surface area and the electron mobility of TiO_2_ photoanode play a key role on the photovoltaic performance of QDSSCs. The electron mobility is defined as the drift velocity of electrons under the driving force of an extra electrical field. Hendry et al. had demonstrated that electron mobility is strongly dependent on the material morphology in nanostructured polar materials due to local field effects [15]. In order to speed up the electron mobility and decrease the possibility of photo-generated charge recombination, 1D nanostructures such as nanotubes [16], nanorods [17, 18], and nanowires [19] were utilized as photoanode for QDSSCs, which supplied direct electrical pathways and facilitate electron transportation; this was considered as a powerful strategy to reduce the electron–hole recombination which abundantly existed in TiO_2_ nanoparticle-based solar cell. Among these 1D architectures, researchers had paid much attention to the rutile TiO_2_ nanorod arrays (NRAs) due to their superior electrical transport performance, excellent chemical stability, high refraction index, and cheap product cost [20–22]. However, it has a vital shortcoming, i.e., small surface area which results in poor QD loading. Thus, the QDSSCs fabricated from the rutile TiO_2_ NRAs exhibited poor photovoltaic performance. A lot of methods have been reported for enlarging the specific surface area of the rutile TiO_2_ NRAs. For example, Lv et al. developed a feasible etching treatment to TiO_2_ NRAs in hydrochloric acid solution under hydrothermal condition, which induced the compact TiO_2_ nanorods split into lots of small nanowires; thus, this method significantly improves the surface area of the TiO_2_ films and in turn, allowed superior dye-loading capacity of the TiO_2_ photoanode, a highest power conversion efficiency (PCE) of 7.91 % was achieved from the DSSCs assembled by the etching TiO_2_ films [23, 24]. From that on, the etching treatment was considered as a powerful strategy for enlarging the inner surface area of the TiO_2_ NRAs. Yuan et al. synthesized long single-crystalline rutile TiO_2_ NRAs with a high surface area by combining a mild hydrothermal method with a chemical etching route, and the DSSCs constructed by 7-h-etched TiO_2_ NRAs exhibited a PCE of 4.69 % [25]. Chen’s group fabricated an ultralong TiO_2_ NRAs (17.6 μm) with a large inner surface area by using a hydrothermal method and post-etched with hydrochloric acid at high temperature. Such TiO_2_ NRAs were utilized as photoanode for CdS/CdSe co-sensitized solar cells and reached a maximum value of 17.22 mA/cm^2^, yielding the highest PCE of 2.66 % [26]. Huang et al. grown polycrystalline TiO_2_ NRAs on FTO substrate and in situ converted NRAs into nanotube arrays (NTAs) by hydrothermal etching. After conversion, more CdSe QDs can be filled in the NTAs, so the PCE of QDSSCs increases by 60 %. QDSSCs with half-etched TiO_2_ nanotubes achieved the best conversion efficiency of 2.44 % [27].

In our work, the moderate etching treatment was introduced to short TiO_2_ NRAs (3.6 μm) and the CdS QDs prepared by successive ionic layer adsorption and reaction (SILAR) was used as the single sensitizer. We focused on the effect of etching time toward QDSSC performance and the underlying reason. Through optimizing the etching time, a PCE of 3.14 % was obtained after TiCl_4_ modifying under the illumination of 100 mW/cm^2^ AM 1.5G solar simulators, this is a relative good performance for CdS-sensitized TiO_2_ NRAs solar cells.

## Methods

### Hydrothermal Synthesis of TiO_2_ NRAs

Hydrothermal synthesis reported by Liu and Aydil was employed for the growth of highly aligned rutile TiO_2_ NRAs [28]. In a typical synthesis, 8 ml deionized water (DI) and 8 ml hydrochloric acid (HCl) with 36.5–38 wt% concentration were mixed together and stirred for 5 min to achieve a homogeneous solution, then 220 μl of titanium butoxide were added into this solution as titanium precursor, followed by another stir until the titanium butoxide was completely dissolved in the hydrochloric acid solution. Finally, the resultant solution was poured into a Teflon-lined container sealed by a stainless steel autoclave (25-ml volume). A piece of FTO glass with a size of 2 cm × 2 cm was used as substrate and leaned on the wall of the reactor with an angle and the conductive side of the FTO substrate faced down. The reactor was transferred into an oven with a temperature at 150 °C maintained for 10 h. While waiting for hydrothermal synthesis to finish and the reactor to cool down to room temperature, the FTO substrate grown by TiO_2_ NRAs was taken out, carefully washed by DI water, and dried using N_2_ gas flow. Finally, the resultant TiO_2_ films were suffered from an annealing at 500 °C for 2 h under air condition.

### Etching Treatment to the TiO_2_ NRAs

Seven milliliters of deionized water was mixed with 9 ml HCl to form an etching solution, then the mixture was transferred into a Teflon-lined container and the TiO_2_ NRAs synthesized by a hydrothermal method were immersed in the etching solution, and the Teflon-lined container was sealed by a stainless steel autoclave. The hydrothermal etching treatment was conducted at 150 °C for 4–6 h. After the autoclave cooled down, the TiO_2_ films on the FTO substrate was taken out from the reactor and immersed in DI water for 2 h to remove the residual acid.

### TiCl_4_ Treatment to the Etching TiO_2_ Films

The etching TiO_2_ films were further modified by TiCl_4_ aqueous solution. In the typical treatment process, the etched TiO_2_ films were immersed in 0.3 M TiCl_4_ aqueous solution at 70 °C for 30 min. After modification, the samples were extensively rinsed with absolute ethanol and followed by 500 °C annealing treatment for 1 h in air.

### Deposition CdS QDs onto TiO_2_ Photoanode

The SILAR method was used for the preparation of CdS QDs on TiO_2_ films. Briefly, the TiO_2_ films treated by hydrochloric acid and TiCl_4_ were immersed in an aqueous solution of Cd(OOCH_3_)_2_ (0.3 M) for 2 min and rinsed with DI water, then immersed in 0.3 M Na_2_S aqueous solution for another 2 min, followed by another rinsing with DI water. Such a SILAR cycle was repeated several times to obtained the desired thickness of the CdS layer. The TiO_2_/CdS photoelectrodes were sintered at 380 °C for 1 h to improve the crystallinity of the CdS QDs

### Characterization

The field-emission scanning electron microscope (FESEM, JEOL JSM-7100F) and transmission electron microscopy (TEM, JEOL JEM 2010) were used for the morphological observation of the samples. The crystalline structure of the products was characterized by X-ray diffractometer (XRD, Brucker D8), the XRD patterns were collected from the samples grown on FTO substrates via θ–2θ scanning mode, and Cu Kα radiation (*λ* = 1.54060 Å) was used as the source operating at 45 kV and 40 mA. The light absorption and the diffused reflectance spectra were examined by UV-vis spectrophotometer (UV-3600, Shimadzu). The Pt films sputtered on an FTO glass substrate was served as the counter electrode and face to face bonded with the TiO_2_/CdS photoelectrode. Polysulfide electrolyte was applied as redox couples and injected into the free space between the two electrodes to complete the QDSSC fabrication. The photocurrent density versus voltage (*J*–*V*) curves of the cells were recorded by digital multi-meter (Keithley 2402) under the illumination of an AM 1.5G solar simulator (Newport, 100 mW/cm^2^), and the irradiated area of devices was defined to be 0.125 cm^2^ using a mask.

## Results and Discussion

**Fig. 1:**
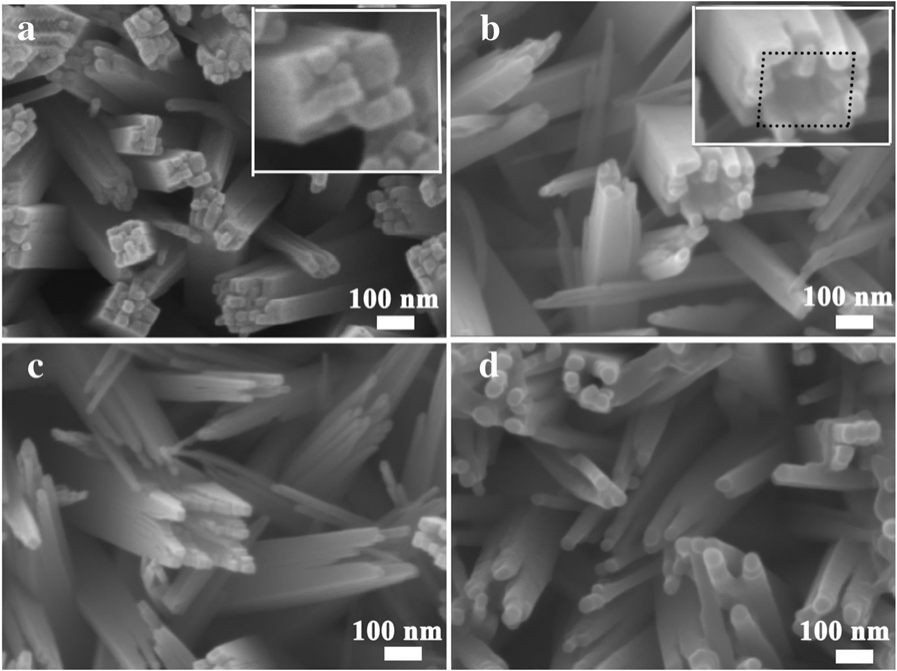
The SEM images of TiO_2_ NRAs before (**a**) and after hydrothermal etched for different times **b** 4, **c** 5, and **d** 6 h

Figure 1 presents the detailed SEM images of as-prepared TiO_2_ NRAs and the nanorods etched for different times. It is obviously seen from Fig. 1a that TiO_2_ nanorods were uniformly distributed on the entire surface of the FTO glass substrate after 10-h hydrothermal synthesis. The nanorods display a geometric shape of tetragonal pillar, which agrees well with the growth preference of tetragonal crystal structure for TiO_2_ nanorod hydrothermal synthesis. The inset in Fig. 1a reveals the detailed top facet of the individual nanorod, lots of step edges can be observed on the top facet of the nanorods, which result from the different growth rates at axial direction among various spots of one nanorod, such step edges provide further substrate for the next growth of the nanorods, whereas the profile of the nanorods display smoothness. Moreover, these nanorods exhibited a large mean diameter about 150 nm, and some adjacent nanorods contacted each other; little space existed among the contacted nanorods, such structure prevented electrolyte to penetrate in and was adverse for solar cell application. So, etching treatment is indispensable for improving the gap space. The as-prepared TiO_2_ NRAs were immersed in a mixed solution that contained 7 ml DI water and 9 ml HCl and suffered from the hydrothermal etching for different times. Figure 1b–d exhibits the morphological character of the TiO_2_ NRAs etched for various times. When etched for 4 h, the center portion of the TiO_2_ nanorods were cut off by the HCl solution, and the nanorods had transformed into a nanocave, which presented an average inner diameter of 120 nm and a wall thickness of 10 nm. The reaction happened in the etching processes could be expressed by the following formula [26, 27].

1 $$ {\mathrm{TiO}}_2\underset{\mathrm{dehydration}}{\overset{\mathrm{etching}}{\rightleftarrows }}\mathrm{T}\mathrm{i}\ \left(\mathrm{I}\mathrm{V}\right)\kern0.5em \mathrm{complex} $$

As the formula expressed, the reaction is reversible, and there are two competing reactions in this system. On the one hand, the TiO_2_ was dissolved in hydrochloric acid solution to produce the Ti (IV) complex. On the other hand, the Ti (IV) complex will hydrolyze into TiO_2_. However, during the etching treatment, the etching solution, contained 9 ml HCl and 7 ml DI water, will push the reaction along the dissolved direction. In addition, the surface stability and reactivity of the TiO_2_ nanorods are dominated by surface chemistry, which is critical for the equilibrium morphology [29]. The surface energy of the rutile TiO_2_ nanorods follows sequences (110) < (100) < (101) < (001) [30, 31]. Generally, the facet with higher surface energy diminishes faster for minimization of the total surface energy. The (001) face corresponds to the core of the TiO_2_ nanorods, and the (110) face is the sidewall of the TiO_2_ nanorods. Thus, the (001) core of the TiO_2_ nanorods is etching faster than the (110) face of the sidewall. As a result, nanocaves appeared on the center portion of the TiO_2_ nanorods by hydrothermal selective etching of the core and the remaining sidewall of (110) face, which can be obviously detected from the inset of Fig. 1b. For the 5-h-etched sample, the inner diameter of the TiO_2_ nanocave continually enlarged. Interestingly, it can be found that the tip wall of the nanocave was divided into lots of small nanowires, and the amount and length of secondary nanowires increased as the etching time extended to 6 h. In order to better understand the etching treatment, the schematic diagrams of the etching process was presented in Additional file 1: Figure S1, images (a), (b), (c), and (d) correspond to the structure of the TiO_2_ films etching for 0, 4, 5, and 6 h, respectively. The inset defines the depth and the inner diameter of the nanocaves. As the Additional file 1: Figure S1 depicted, when the etching time was 4 h, the center portion of the nanorods was cut off by hydrochloric acid to form a nanocave. As the etching time was prolonged to 5 h, the upper part of the nanocave wall would split into small nanowires. Extending the etching time, the length and the amount of the secondary nanowires were continually increased.

**Fig. 2:**
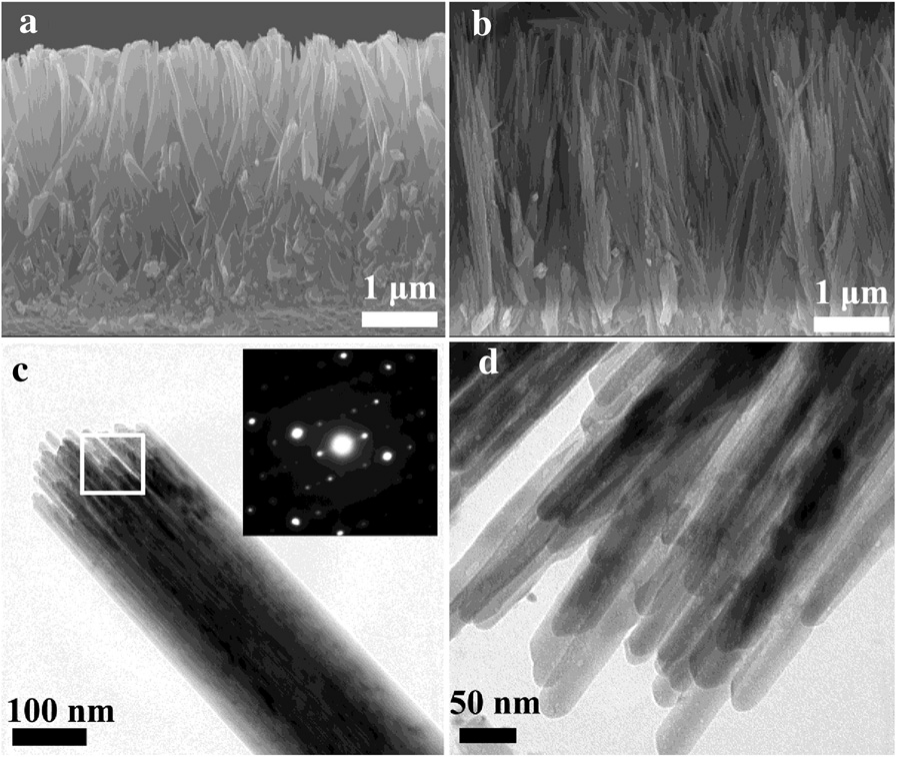
The cross-sectional images of TiO_2_ NRAs before (**a**) and after 6-h etching treatment (**b**). The typical TEM images (**c**) and SAED pattern (*inset*) of an individual TiO_2_ nanorod with 6-h etching treatment, enlarged areas marked by *white rectangular frame* is presented in (**d**)

Figure 2a, b reveals the cross-sectional views of the TiO_2_ NRAs before and after the 6-h etching treatment. For the as-prepared sample, the thickness of the nanorod films was 3.6 μm, and the nanorods display high density; there is a little space between the adjacent nanorods especially for the bottom part of the arrays, which is well consistent with the result of Fig. 1a. As shown in Fig. 2b, the NRAs after 6-h etching exhibited almost the identical thickness with the original sample. Furthermore, the hydrothermal etching lead the virgin TiO_2_ nanorod to split into lots of small nanowires, and the compact NRAs became loose. Thus, the interstitial space between the adjacent nanorods dramatically amplified especially for the upper half part of the TiO_2_ NRAs, resulting in a large surface area for the TiO_2_ NRAs. The further detailed morphology of the 6-h etching samples was detected by TEM. From Fig. 2c, d, it can be obviously seen that the tip part of the nanorod was divided into lots of small nanowire with a diameter of 20 nm. The selected area electron diffraction (SAED) pattern revealed that the nanorods was single-crystal TiO_2_, and such structure displays unique advantage in QDSSC application, because they can provide high-speed pathway for electron transport with few crystal boundary.

**Fig. 3:**
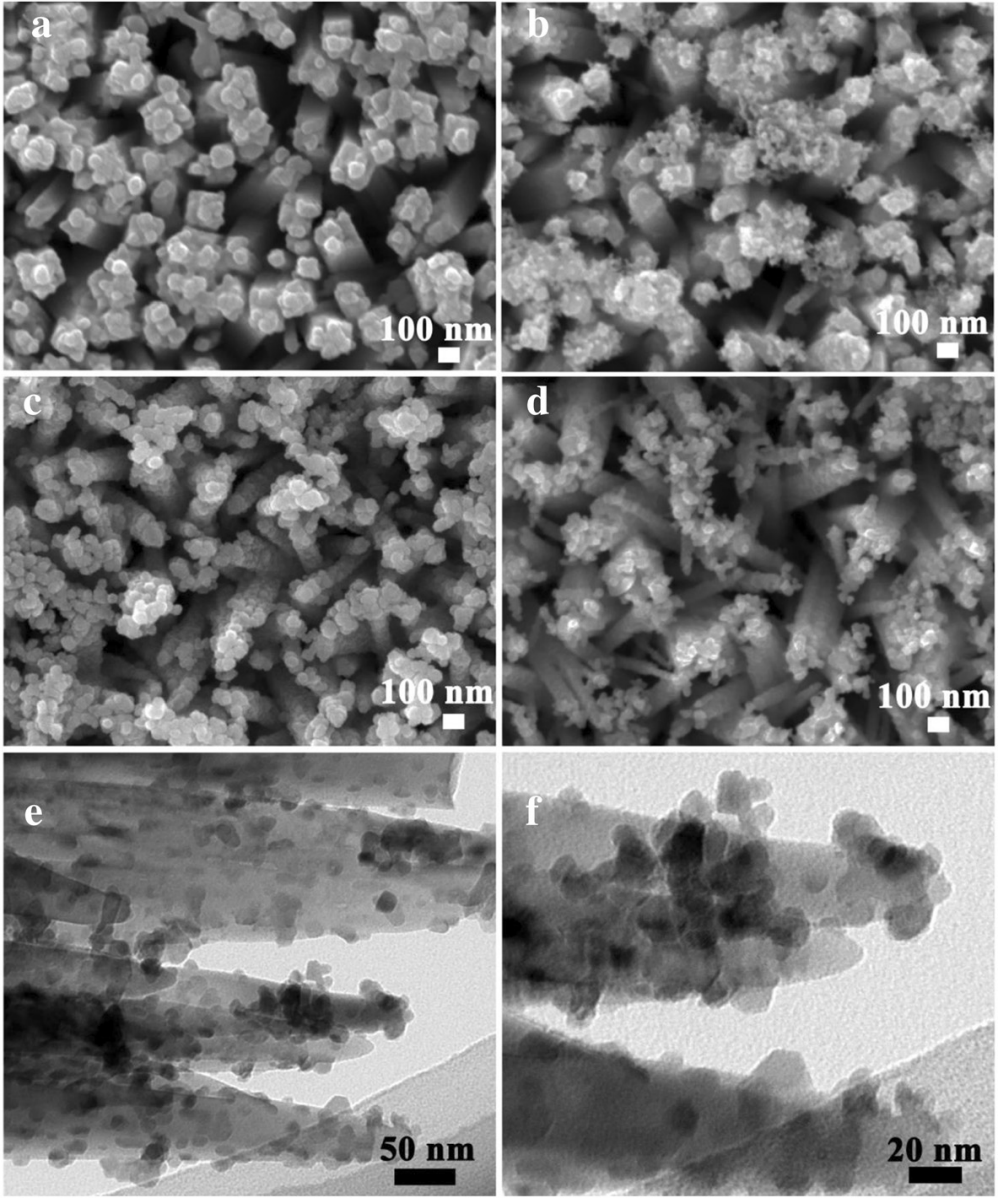
The top views of CdS QD-sensitized TiO_2_ NRAs which was etched for different times **a** 0, **b** 4, **c** 5, and **d** 6 h. **e** and **f** give the typical TEM images of CdS-sensitized 6-h-etched TiO_2_ NRAs

Figure 3 shows the top views of the TiO_2_ films sensitized by CdS QDs. It can be seen from Fig. 3a that a plentiful of CdS nanoparticles were covered on the surface of the as-prepared TiO_2_ nanorods to form TiO_2_/CdS nanocable. In addition, the side and top faces of the nanorods were roughened by CdS nanocrystal, which may be in favor of the incident light absorption and diffused reflection. The worth noting point was that the TiO_2_/CdS nanocable became very compact after CdS coating, a little free space was left in the films, it would prevent the electrolyte to penetrate into and blocked the CdS sensitizer contact with electrolyte, and this was adverse for the PCE of the assembled QDSSCs. For the 4-h etching sample, the inner and outer surfaces of the nanocave were attached by CdS QDs, and the amount of QDs was obviously greater than that of un-etching sample. Moreover, it can be found that the gap space between the adjacent nanocables became large, which was in favor of QDSSC utilization. When etching duration prolonged to 5 h, the wall of the nanocaves was further split into lots of small nanowires, and all surfaces of the small nanowires can provide sites for CdS QD adsorption, which results in the enhancement of QD loading. In addition, the amount of QDs deposited on the TiO_2_ films further increased when the etching time extended to 6 h, because the length and the amount of the secondary nanowires continually increased as shown in Fig. 1d. The typical TEM images of CdS-sensitized 6-h-etched TiO_2_ NRAs have been provided in Fig. 3e, f. From the images, we can see that the CdS sensitizers sufficiently cover the secondary nanowires, the CdS nanoparticles are faceted with occasionally irregular shapes, and the particles presents a size ranges from 5 to 15 nm.

**Fig. 4:**
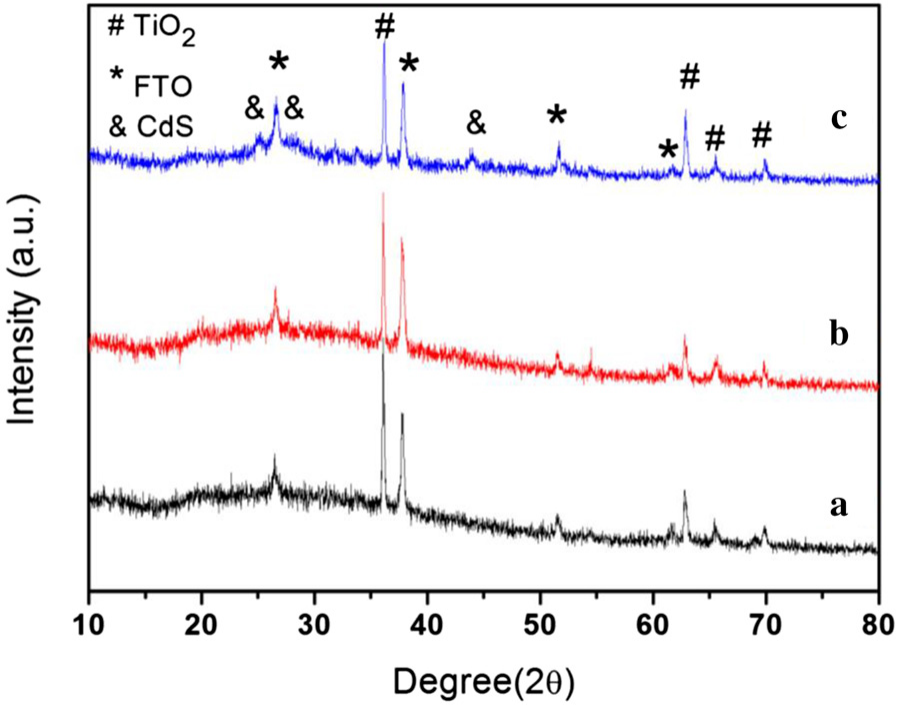
The XRD patterns of TiO_2_ NRAs, TiO_2_ NRAs etching for 5 h, and TiO_2_ NRAs sensitized by CdS QDs

The microstructure of the TiO_2_ NRAs before and after the etching treatment are presented in Fig. 4a, b characterized by an XRD instrument. For the FTO/TiO_2_ un-treated sample, except for the peaks from the FTO substrate, the diffraction peaks located at 36.2° and 62.8° can be indexed to the (101) and (002) planes of tetragonal rutile TiO_2_ (PCPDF No.89-4920), and the (101) peak exhibited the strongest intensity. The similar result had also been detected in other literatures [32–34]. For the rutile TiO_2_ powder containing randomly oriented crystals, the most intense diffraction peak should be the (110) (the reference data in JCPDS 89-4920) which was similarly observed for the rod-shaped rutile TiO_2_ nanoparticles [35]. However, in our case, the (101) peak presents the highest diffraction intensity, whereas the (110) peak intensity is noticeably weak. The highly intense (101) peak along with the enhanced (002) peak in the NRA film indicates that the rutile crystal grows with (101) plane parallel to the FTO substrate and the nanorods are oriented along the [002] direction. Comparing the XRD patterns of the TiO_2_ films before and after the etching treatment, we can found that both of the two samples exhibited the identical diffraction peaks position regardless of the intensity difference, demonstrating that the etching treatment in hydrochloric acid have not damaged the rutile crystal structure of the TiO_2_ films. The rutile TiO_2_ exhibits some advantages over anatase such as higher chemical stability, higher refractive index, and cheaper production cost. Besides these advantages, TiO_2_ NRAs with rutile phase has approximately 100 times larger electron mobility than nanoparticles TiO_2_, which results from its one-dimensional structure, highly crystalline and defect-free. Thus, the TiO_2_ NRAs with a rutile phase exhibits unique superiority in the application of sensitized solar cell due to its lower electrical transport resistance, and this was in favor of reducing the electron recombination rate [15, 17, 36]. The XRD patterns of FTO/TiO_2_/CdS are shown in Fig. 4c, and besides the peaks indexed to SnO_2_ and TiO_2_, the other three peaks appeared at 25.1°, 28.4°, and 43.9° which matched well with the (100), (101), and (110) planes of hexagonal CdS (JCPDS no. 06-0314), respectively. Moreover, the full width at half maximum (FWHM) of the CdS diffraction peaks are broad, suggesting the small particle size of CdS QDs deduced by Debye–Scherrer equation.

**Fig. 5:**
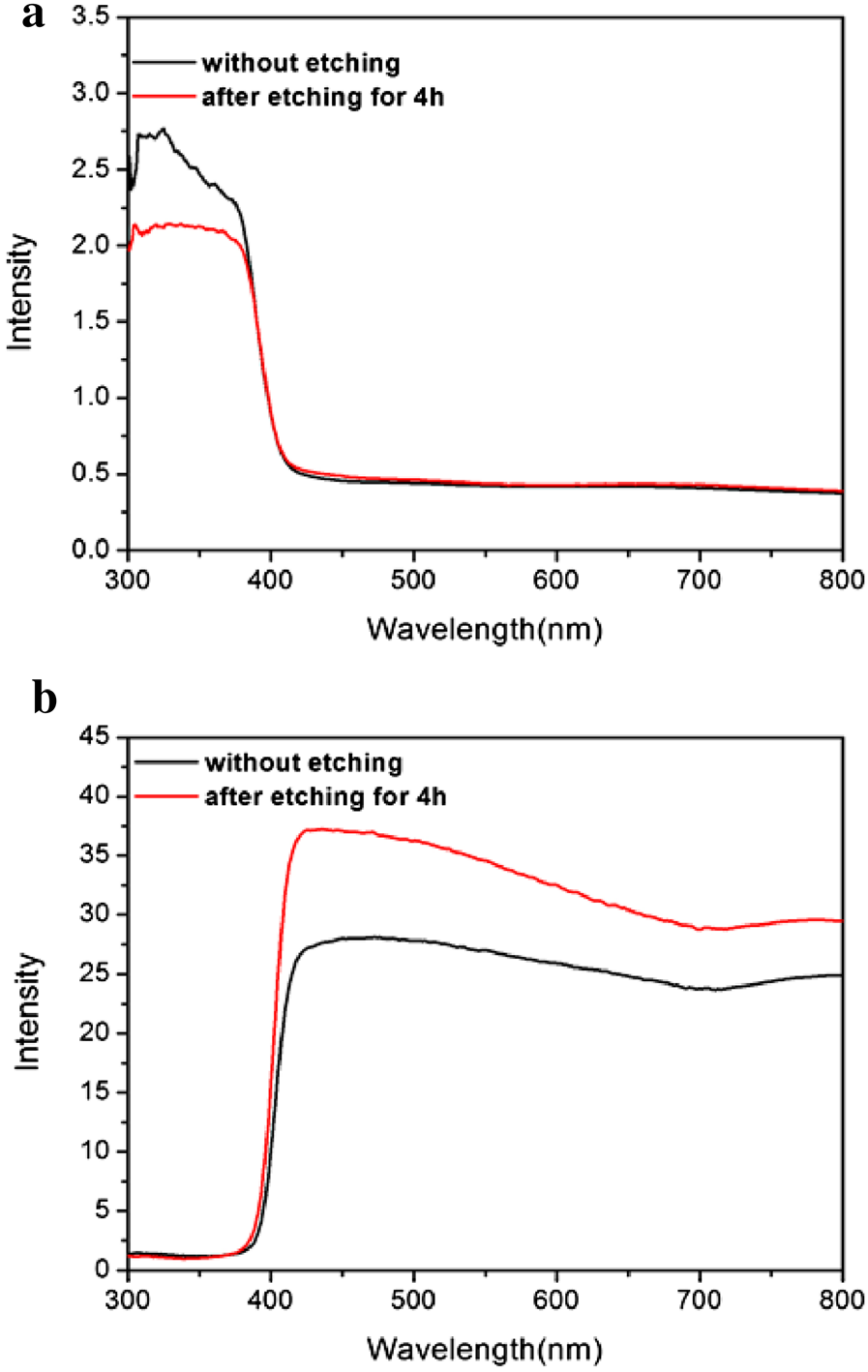
The absorption spectra (**a**) and diffused reflectance curves (**b**) of the TiO_2_ nanorod before and after hydrothermal etching

Figure 5a gives the Uv-vis absorption curves of the TiO_2_ films before and after hydrothermal etching. For the un-etching sample, a steep UV absorption edge occurs at ~410 nm, and TiO_2_ displays no absorption in the visible light range due to its large band gap of 3.2 eV. Comparing the light absorption curves before and after the etching treatment, it can be found that the two samples exhibited the same absorption edge, because the etching treatment does not change the rutile crystal structure of TiO_2_ nanorod as the XRD result shown. In addition, the light-scattering capacity of the photoanode has significant influence on the light-harvesting performance of the photoelectrode (photoanode sensitized by QDs). When the incident light irradiates on the photoelectrode, the photoanode with superior light-reflectance ability should has a high probability for capturing the incident light, this can helps to improve the short-circuit current density (*J*_sc_). Usually, the light-scattering ability can be characterized by diffused reflectance spectra. Figure 5b shows the diffused reflectance spectra of the un-treated and 5-h etching TiO_2_ films, and it can be found that the reflectance index of the etching sample was higher than that of the un-etching sample; this can be explained by the relatively random structure after the etching treatment, such random structure can provide more light-scatter points [37, 38].

**Fig. 6:**
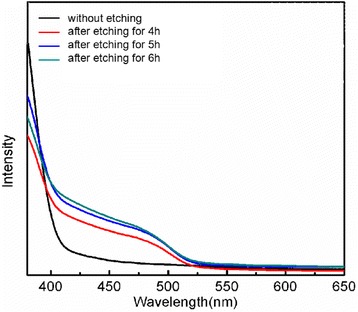
Uv-vis absorption spectra of FTO/TiO_2_/CdS photoelectrodes etched for different times

Figure 6 reveals the typical optical absorbance spectra of the TiO_2_/CdS photoelectrodes with different etching time. In comparison with the pure TiO_2_ photoanode, the light absorbance of TiO_2_/CdS photoelectrodes exhibited significant enhancement in visible light region, suggesting that the CdS QD was an efficient photo-sensitizer for QDSSC utilization. Moreover, the additional absorption edge emerged at 525 nm for TiO_2_/CdS photoelectrodes corresponding to the CdS band gap of 2.36 eV. In addition, it can be found that the absorbance intensity in visible light region enhanced with the etching duration, this may be due to the increase of CdS nanoparticles amount attached on the TiO_2_ photoanode surface as shown in Fig. 3.

**Fig. 7:**
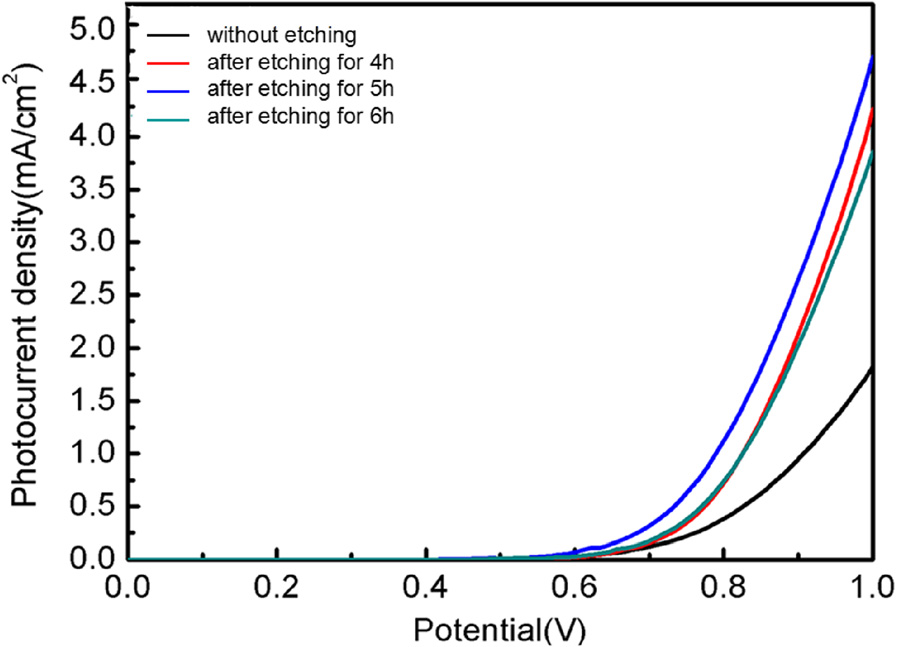
Current density versus potential curves for FTO/TiO_2_/CdS photoelectrodes etched with various times measured in the dark

The TiO_2_/CdS photoelectrodes with different etching times were face to face bonded with FTO/Pt counter electrodes to assemble QDSSCs, and the sulfide-based electrolyte was used as redox couples to maintain the photo-sensitizer electrical neutrality. Figure 7 shows the dark *J*–*V* curves of QDSSCs constructed from the TiO_2_ films with different etching times. All the curves indicated typical rectifying behavior as the diode property. When the extra voltage was applied between the photoelectrode and counter electrode, the intrinsic electron in the CdS QDs would transport along a certain direction under the force of electrical field, which caused the dark current. The magnitude of the dark current can be used to estimate the charge recombination [39]. In general, the electron recombination reaction involved two processes: the photoinjected electrons in TiO_2_ conduction band recombined with the oxidized QDs and/or with S_x_^2−^ in the electrolyte. Because the regeneration of the QDs by S^2−^ is remarkably faster than the charge transfer from TiO_2_ to the oxidized QDs, the recombination between photo-generated electron and the oxidized QDs is negligible. Thus, the dark current usually represented the recombination between S_x_^2−^ ions and the photo-generated electrons in the semiconductor [40]. The etching photoanode displays a higher dark current compared with the un-etching samples, indicating more charge recombination in the etching samples, and this may be attributed to the enhancement of electron trapping sites after etching treatment.

**Fig. 8:**
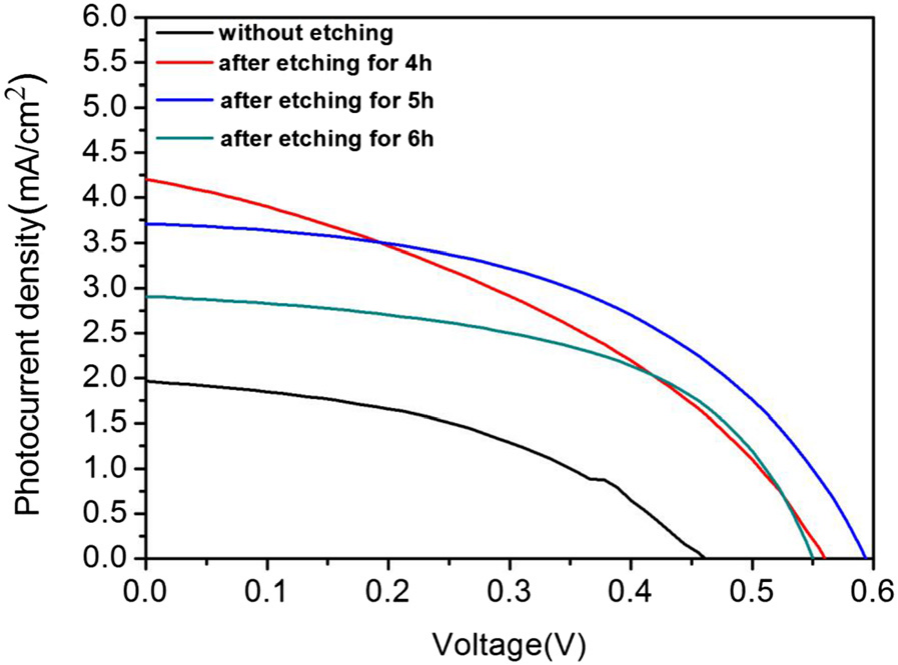
J–V curves of QDSSCS based on TiO_2_ photoanode etching for different times under the illumination of AM 1.5G light at 100 mW/cm^2^. The active area was 0.125 cm^2^

Figure 8 presents the photocurrent density-voltage performance of QDSSCs made from TiO_2_/CdS photoelectrodes with various etching times. The detail photovoltaic parameter of the QDSSCs including short-circuit current density (*J*_sc_), open-circuit voltage (*V*_oc_), fill factor (FF), and power conversion efficiency (*η*), are listed in Table 1. As shown in Table 1, the cell constructed by un-treated TiO_2_ NRAs gives a *J*_sc_ of 1.96 mA/cm^2^, an *V*_oc_ of 0.45 V, and a FF of 42.9 %, yielding a *η* of 0.38 %. Through the etching treatment, the cells exhibit remarkable increase in *J*_sc_ and *V*_oc_ compared with that of the un-treated sample, and these results are mainly contributed by the enhanced QD loading ability, which ascribes to the enlarged surface area after hydrothermal etching. In addition, the PCE initially increases with etching time then decreases. A maximum PCE of 1.07 % is obtained when the etching time is 5 h. As the SEM analysis, the amount of QD loading increased with the etching duration, which helps to strengthen the visible light absorption. Therefore, the photo-generate current density increases when the etching time increases from 4 to 5 h, yielding an improvement in PCE. As the etching time continually extended to 6 h, the interface between TiO_2_ nanorods and FTO substrate will become unstable after long-term corrosion, because the TiO_2_ nanoparticle in the interfaces will gradually dissolve in acid solution, and the interface resistance increases, which results in the decrease of the PCE afterwards.

**Table 1:** Photovoltaic parameters obtained from the *J*–*V* curves of QDSSCs based on TiO_2_ photoanodes etched for different times

Samples	*V*_oc_ (V)	*J*_sc_ (mA/cm^2^)	Fill factor (%)	Efficiency (%)
None	0.45	1.96	42.9	0.38
4 h	0.55	4.15	38.9	0.90
5 h	0.59	3.68	49.4	1.07
6 h	0.55	2.89	53.7	0.85

**Fig. 9:**
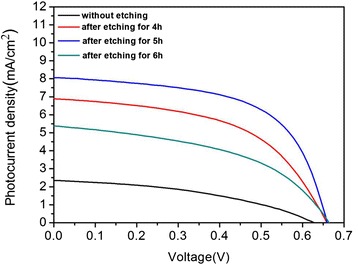
J–V characteristics of QDSSCS fabricated from the etching TiO_2_ NRAs with TiCl_4_ treatment

The titanium tetrachloride (TiCl_4_) treatment is considered as an effective route for improving the photovoltaic properties of QDSSCs. In this work, the TiCl_4_ modification was carried out to the TiO_2_ films with different etching times. As the typical modification process, the TiO_2_ films etched for various times were immersed in 0.3 M TiCl_4_ aqueous solution at 70 °C for 30 min. After the treatment, the samples were taken out from the TiCl_4_ aqueous solution, extensively rinsed with absolute ethanol, and then annealed at 500 °C in air atmosphere for 1 h. Additional file 1: Figure S2 shows the SEM image of the TiO_2_ NRAs modified with 0.3 M TiCl_4_ at 70 °C for 30 min. It can be observed that the side face of the NRAs was attached with lots of TiO_2_ nanoparticles after TiCl_4_ treatment, which roughened the surface, and the size of the nanoparticle was 2 nm. Figure 9 reveals the *J*–*V* curves of the QDSSCs assembled by the TiO_2_ photoanode treated with TiCl_4_ under the illumination of AM 1.5 solar simulators (100 mW/cm^2^), and the corresponding parameters of photovoltaic performance are deduced and summarized in Table 2. As shown in Table 2, the cell fabricated from the un-etching TiO_2_ NRAs treated with TiCl_4_ gives a *J*_sc_ of 2.33 mA/cm^2^, an *V*_oc_ of 0.61 V, a FF of 41.4 %, and a *η* of 0.59 %. In addition, it is worth noting that the TiCl_4_ treatment gives rise to the higher *J*_sc_ values than that of the un-treating sample. A highest PCE of 3.14 % is achieved after TiCl_4_ treatment. As the SEM in Additional file 1: Figure S2 shown, after TiCl_4_ modification, a lot of extra TiO_2_ nanoparticles were synthesized on the surface of the prepared TiO_2_ nanostructure, which lead to the further increase of TiO_2_ specific surface area. Consequently, the QD loading ability of the photoanode was strengthened. Moreover, a thin TiO_2_ layer was covered on the bare surface of FTO substrate after TiCl_4_ modification, which can block the recombination between electrons in photoanode and positive charge in electrolyte [41, 42]. In addition, TiCl_4_ modification can also improve the light-scattering capacity of the TiO_2_ films. All these functions of TiCl_4_ treatment resulted in high energy conversion.

**Table 2:** Photovoltaic parameters obtained from the *J*–*V* curves of QDSSCs based on TiCl_4_ treatment of TiO_2_ photoanodes etched for different times

Samples	*V*_oc_ (V)	*J*_sc_ (mA/cm^2^)	Fill factor (%)	Efficiency (%)
None	0.61	2.33	41.4	0.59
4 h	0.65	6.86	52.4	2.36
5 h	0.65	8.06	59.2	3.14
6 h	0.66	5.36	47.3	1.68

## Conclusions

In this study, a hydrothermal method was used to grow TiO_2_ NRAs on FTO substrate. For the sake of a large specific surface area, a facile hydrothermal etching was employed to the TiO_2_ NRAs. The relation between the etching time and the performance of TiO_2_ films had been comprehensively studied. The results showed that the etching treatment enlarged the gap space among the compact nanorods and hollowed out the center part of the nanorod to form a nanocave, and the wall of the nanocave split into lots of small nanowires; these changes in morphology lead to the improvement of the surface area. In addition, the hydrothermal etching in HCl solution did not damage the rutile crystal structure of the TiO_2_ nanorods and enhanced the diffused reflectance ability of photoanode. All these factors resulted in better photovoltaic performance for the QDSSCs made from the etching TiO_2_ films. Finally, through modifying with TiCl_4_, a relatively high PCE of 3.14 % is obtained after optimizing the etching time.

## Additional file

Additional file 1: Figure S1. The schematic diagrams of the etching process. Images (a), (b), (c), and (d) correspond to the structure of TiO_2_ films etching for 0, 4, 5, and 6 h. The inset defines the depth and the inner diameter of the caves. **Figure S2.** The SEM images of the TiO_2_ NRAs modified with 0.3 M TiCl_4_ at 70 ^o^C for 30 min.
