# Supplementary material for: Hydrothermal Etching Treatment to Rutile TiO2 Nanorod Arrays for Improving the Efficiency of CdS-Sensitized TiO2 Solar Cells
Source: Nanoscale Res Lett. 2016 Jan 12;11:12. doi: 10.1186/s11671-016-1236-9 (PMC4709343; doi:10.1186/s11671-016-1236-9)
Supplement: Additional file 1: Figure S1. — The schematic diagrams of the etching process. Images (a), (b), (c), and (d) correspond to the structure of TiO2 films etching for 0, 4, 5, and 6 h. The inset defines the depth and the inner diameter of the caves. Figure S2. The SEM images of the TiO2 NRAs modified with 0.3 M TiCl4 at 70 oC for 30 min. [file 11671_2016_1236_MOESM1_ESM.doc]

**Hydrothermal Etching Treatment to Rutile TiO_2_ Nanorod Arrays for Improving The Efficiency of CdS Sensitized TiO_2_ Solar Cells.**

**Supporting Information**

**Jingshu Wan, Yuzhu Tong, Shuhuang Chen, Yunxia Hu, Baoyuan Wang*****, Yang Xu, Rong Liu, Hao Wang***

Hubei Collaborative Innovation Center for Advanced Organic Chemical Materials, Faculty of Physics and Electronic Science, Hubei University, Wuhan 430062, PR China

*correspondence author. Email: [*baoyuanw@163.com*](mailto:baoyuanw@163.com) (B.Y. Wang); [*nanoguy@126.com*](mailto:nanoguy@126.com) (H. Wang)


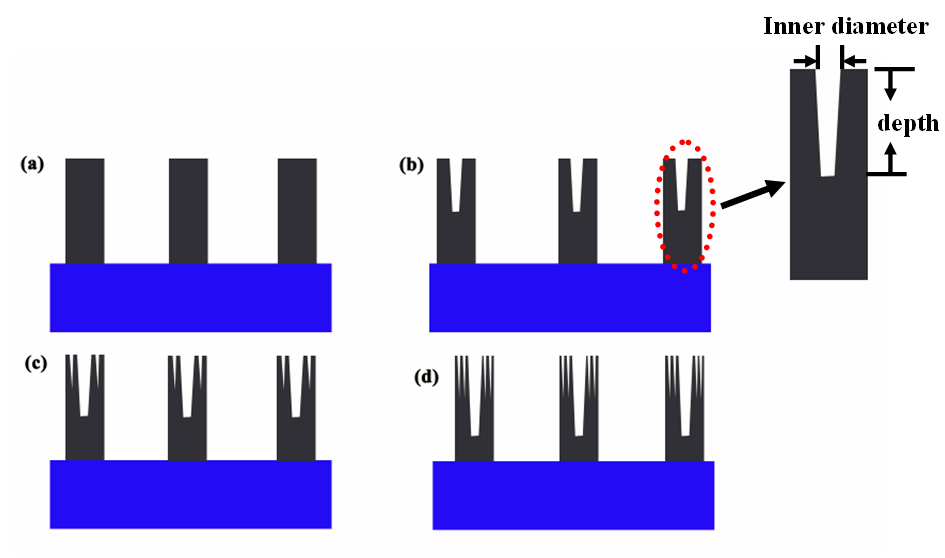


Figure 1s the schematic diagrams of etching process. Images (a), (b), (c) and (d) correspond to the structure of TiO_2_ films etching for 0 h, 4 h, 5 h and 6 h., the inset defines the depth and the inner diameter of caves.


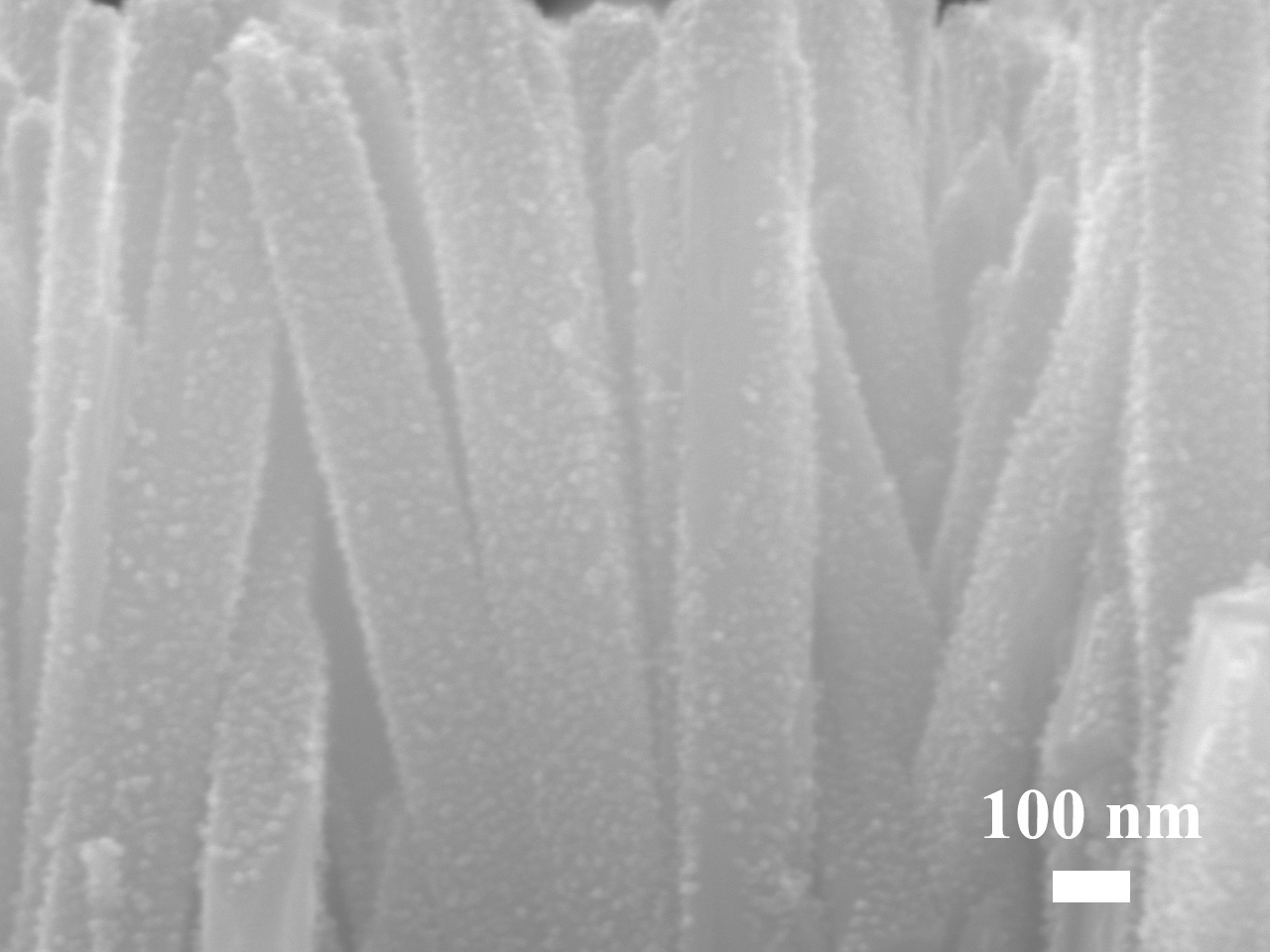


Figure 2s The SEM images of the TiO_2_ NRAs modified with 0.3 M TiCl_4_ at 70 ^o^C for 30 min.
